# A rare case of bladder cancer that metastasized to brain, heart, and lung lymph nodes benefited from immunotherapy

**DOI:** 10.1186/s12957-022-02876-9

**Published:** 2022-12-19

**Authors:** Lian-kai Zhu, Zhong-jian Li, Zhi-bo Wang, Jin-tao Chen, Hua-jun Zhang, Xu-wei Zhao, Hong-yao Liu

**Affiliations:** Third Hospital of Shanxi Medical University, Shanxi Bethune Hospital, Shanxi Academy of Medical Sciences, Tongji Shanxi Hospital, Taiyuan, 030032 China

**Keywords:** Bladder cancer, Brain metastasis, Heart metastasis, Immunotherapy, Tislelizumab

## Abstract

Bladder cancer is a common malignant tumor of the genitourinary system, with the primary cause of death being metastasis. The most common metastatic sites are the lymph nodes, liver, lung, bone, peritoneum, pleura, kidney, adrenal gland, and the intestine. Brain and heart metastases are rare. In this report, we describe a patient who had pulmonary lymph node metastases more than a year after being diagnosed with bladder cancer, followed by brain and cardiac metastases more than two years later. Following the failure of standard first-line chemotherapy, the patient accepted 6 cycles of tislelizumab immunotherapy. The re-examination revealed that the bilateral frontal brain metastases had vanished, the right temporal lobe metastases had been greatly decreased, the neurological symptoms had been alleviated, and the cardiac metastases had disappeared. This is a rare clinical case with encouraging effects of tislelizumab and can serve as a model for the treatment of similar patients.

## Introduction

Bladder cancer ranks 13th in terms of malignant tumor incidence in China, with a crude incidence rate of 5.80/100,000 and a crude mortality rate of 2.37/100,000 [1]. The primary cause of death from bladder cancer is metastasis. The 5-year relative survival rate is 70% for patients with tumors confined to the bladder but drops to 34% for patients with diseases that spread locally beyond the bladder and to 5% for patients with distant metastases [2]. By reviewing autopsy results of 367 patients with pT2–4 bladder cancer, Wallmeroth et al. revealed that metastases were found in 251 of 367 patients (68%), with the most common sites of metastases being regional lymph nodes (90%), liver (47%), lung (45%), bone (32%), peritoneum (19%), pleura (16%), kidney (14%), adrenal gland (14%), and the intestine (13%) [3]. Brain metastases from bladder cancer occur at a rate of 0 to 7% [4], while cardiac metastases have not been clinically recorded. The primary goal of treatment for these patients is to alleviate symptoms and avoid tumor recurrence. Currently, the most common treatment options for brain metastases are surgery, systemic chemotherapy, and whole-brain radiation or stereotactic external radiation [5], whereas heart metastases remain untreated on a regular basis. However, patients with brain metastases have a poor prognosis regardless of treatment, with a median survival time of only 2–4 months [6, 7], and patients with heart metastases also have a poor prognosis.

Immunotherapy for bladder cancer has advanced rapidly in recent years. The discovery of immune checkpoint inhibitors (ICIs) such as programmed death 1 (PD-1) antibodies, in particular, has resulted in a significant breakthrough [8]. Previous studies have demonstrated that second-line ICI treatment can significantly improve patient survival and may even be more effective in patients with high PD-L1 expression [9–13]. However, as for advanced urothelial cancer, Martini et.al. discovered no OS benefit for patients treated with first-line ICI versus chemotherapy in the overall population, cisplatin-ineligible patients, and PD-L1-high patients [14]. Tislelizumab (BeiGene Co., Ltd., Beijing, China), a humanized IgG4 monoclonal antibody with high affinity and specificity for PD-1, was approved by China’s National Medical Products Administration (NMPA) in 2020 for the treatment of locally advanced or metastatic urothelial carcinoma in patients who have failed to respond to first-line chemotherapy [15]. In this report, we present a 64-year-old man with bladder cancer who had metastases to the brain, heart, and lung lymph nodes. Following the 6 cycles of tislelizumab immunotherapy, the bilateral frontal brain metastases had vanished, the right temporal lobe metastases had been greatly decreased, and the cardiac metastases had disappeared. The patient received 6 cycles of tislelizumab immunotherapy over the next 5 months, with his condition remaining stable. At the time this article was written, it had been 16 months since the patient was diagnosed with brain metastasis and heart metastasis of bladder cancer, his daily life quality has not been affected except for an occasional mild headache.

## Case presentation

**Fig. 1:**
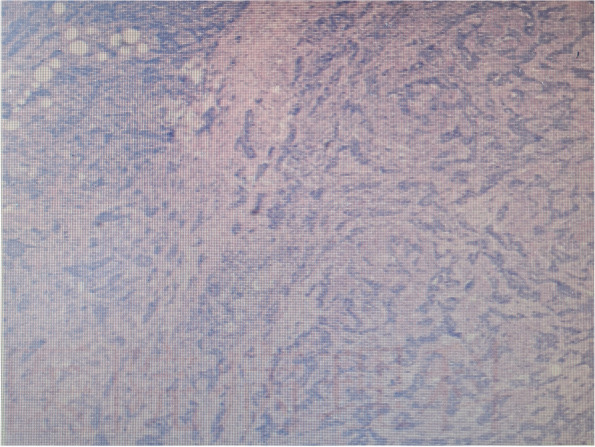
The HE staining of TURBT biopsy indicated high-grade urothelial carcinoma. (TCC G3 pT1)

In January 2019, a 64-year-old male was referred to a nearby hospital for cystoscopy due to “gross hematuria” and the results indicated a “bladder mass.” The transurethral resection of bladder tumor (TURBT) was conducted, and BCG perfusion therapy was followed, with the pathological diagnosis of high-grade non-muscle invasive bladder urothelial carcinoma. In August 2019, cystoscopy revealed recurrence of the bladder tumor, and TURBT was administered with the same pathological findings (TCC G3 pT1) (Fig. 1). Following surgery, 30 mg pirarubicin was infused into the bladder once a week for the first 2 months and once a month for the next treatment. During the reexamination in April 2020, a CT scan and pathological biopsy performed via cystoscopy of the urinary system indicated tumor relapse. Chest CT revealed enlarged lymph nodes beneath the right hilar and mediastinal carina, indicating bladder cancer distant metastasis following surgery. From April 2020 to June 2020, the patient received 3 cycles of gemcitabine and cisplatin (GC) regimen systemic chemotherapy. Adverse reactions such as severe dizziness, nausea, and vomiting occurred during chemotherapy, interfering with normal life and remaining unresolved significantly after symptomatic treatment.

**Fig. 2:**
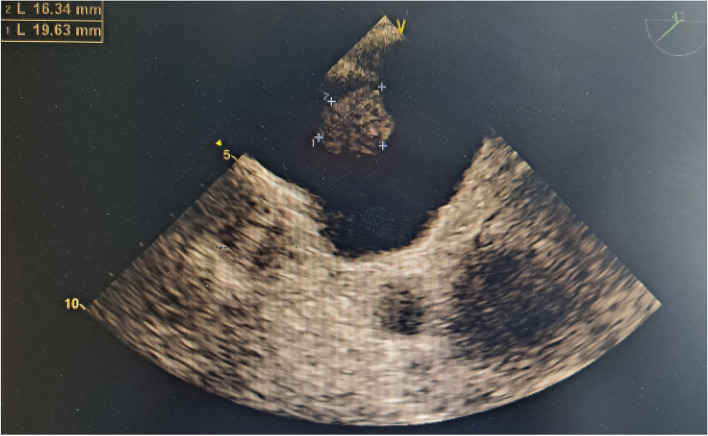
The transesophageal ultrasonography in April 2021. A solid space-occupying lesion in the left atrium at the size of 1.9 cm × 1.6 cm

**Fig. 3:**
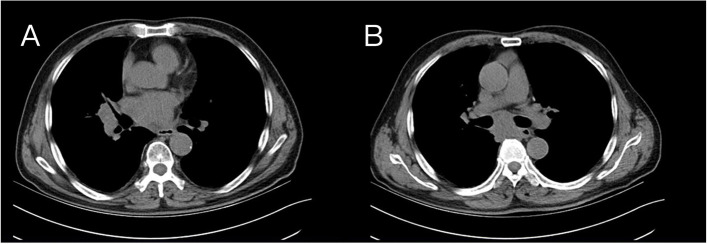
The chest CT imaging of lung metastasis in April 2021 before tislelizumab administration. **A** The enlargement of the right hilar lymph nodes. **B** The swollen subcarinal lymph nodes in the left upper lobe

**Fig. 4:**
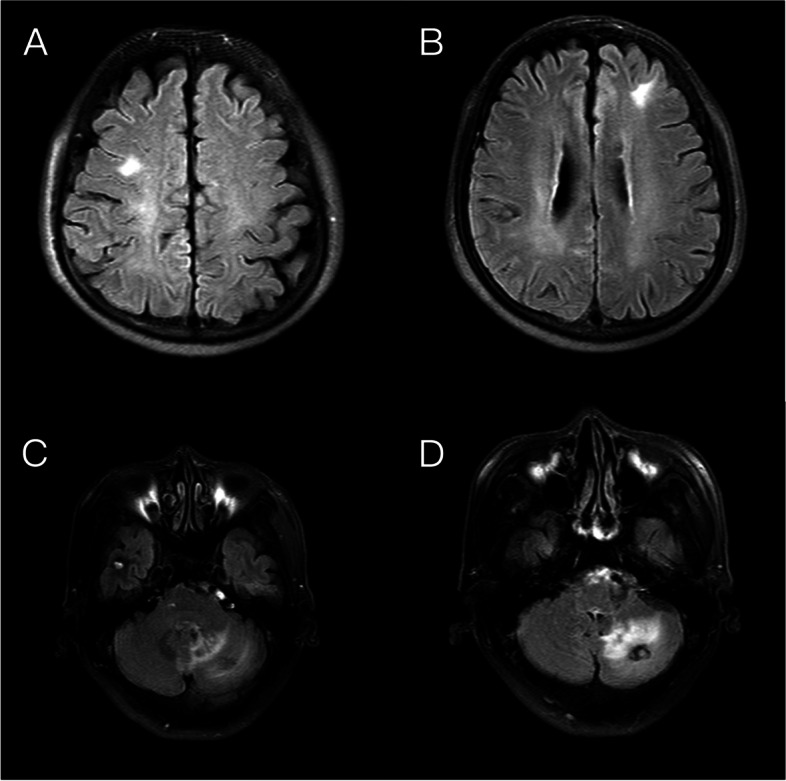
The brain MRI prior to tislelizumab administration in April 2021. Multiple metastasis lesions in the right frontal lobe (**A**), left frontal lobe (**B**), right temporal lobe (**C**), and left cerebellar hemisphere (**D**)

The patient was readmitted to the hospital in October 2020 due to gross hematuria. CT of the urinary system revealed that the bladder lesion remained unchanged from April 2020; however, chest CT revealed that the subcarinal lymph nodes in the mediastinum had grown in size since April 2020. The TURBT-mediated pathological examination revealed a high-grade invasive urothelial carcinoma with adenoid differentiation, infiltrating smooth muscle tissue, with no obvious vascular or nerve complication (TCC G3 pT2). Following that, the patient received 3 cycles of systemic chemotherapy with the standard GC regimen and pirarubicin bladder infusion chemotherapy from October to December 2020. In April 2021, the patient was admitted to the hospital after experiencing dizziness, headache, and palpitations. Electrocardiography revealed atrial fibrillation with a rapid ventricular rate, while transesophageal ultrasonography indicated a solid space-occupying lesion in the left atrium at the size of 1.9 cm × 1.6 cm (Fig. 2). Based on the patient’s medical history and cardiac surgical consultation, we speculated that the solid space-occupying lesion in the left atrium was the metastases from bladder cancer. However, patients and their families decline biopsy to further elucidate the mass’ origins. Enlarged lymph nodes could be seen beneath the right hilar and mediastinal carina on chest CT, indicating pulmonary lymph nodes metastasis (Fig. 3). Multiple metastases in the right temporal lobe and frontal lobe, left frontal lobe, and cerebellar hemisphere were detected using MRI, with the largest measuring approximately 2.04 cm × 1.64 cm × 1.80 cm (Fig. 4). The patient denied dysarthria, vision changes, cognitive changes, or difficulty walking.

**Fig. 5:**
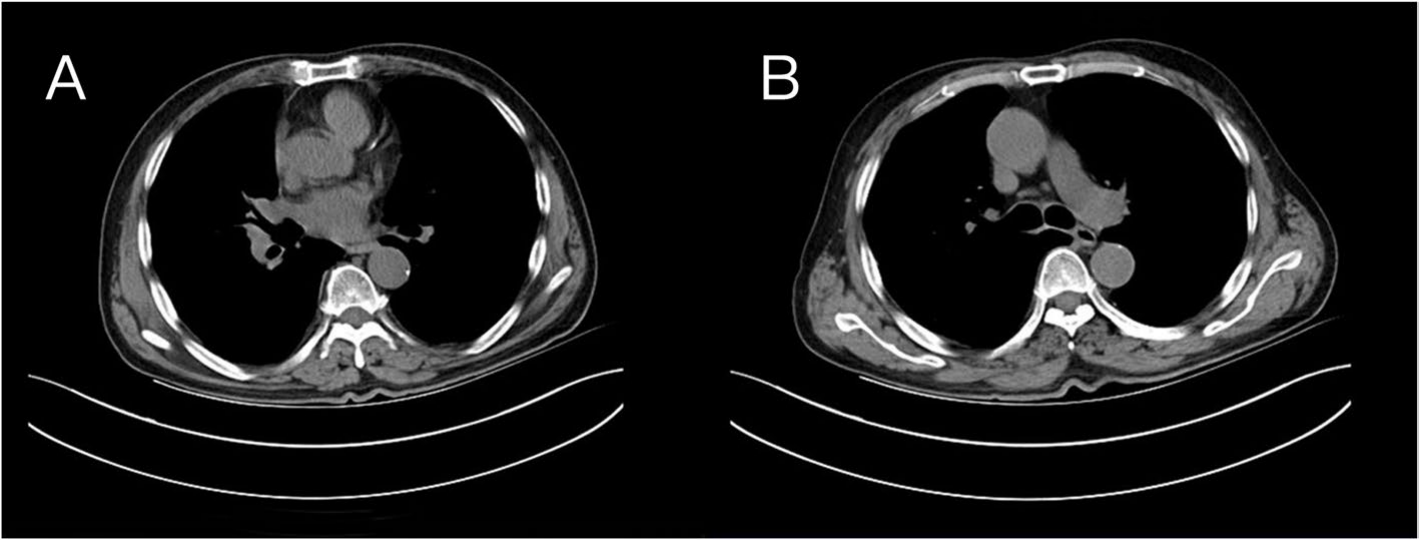
The chest CT imaging after tislelizumab therapy in August 2021.In comparison to that of April 2021, the volume of parabronchial soft tissue masses in the right lung's middle lobe was significantly reduced (**A**), and enlarged lymph nodes beneath the tracheal carina was significantly reduced (**B**)

**Fig. 6:**
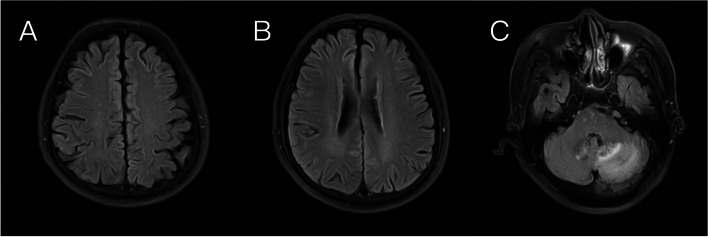
The brain MRI after tislelizumab therapy in August 2021. **A, B** Compared to that of April 2021, the lesions in the bilateral frontal lobe vanished. **C** The lesions in the right temporal lobe were significantly reduced

**Fig. 7:**
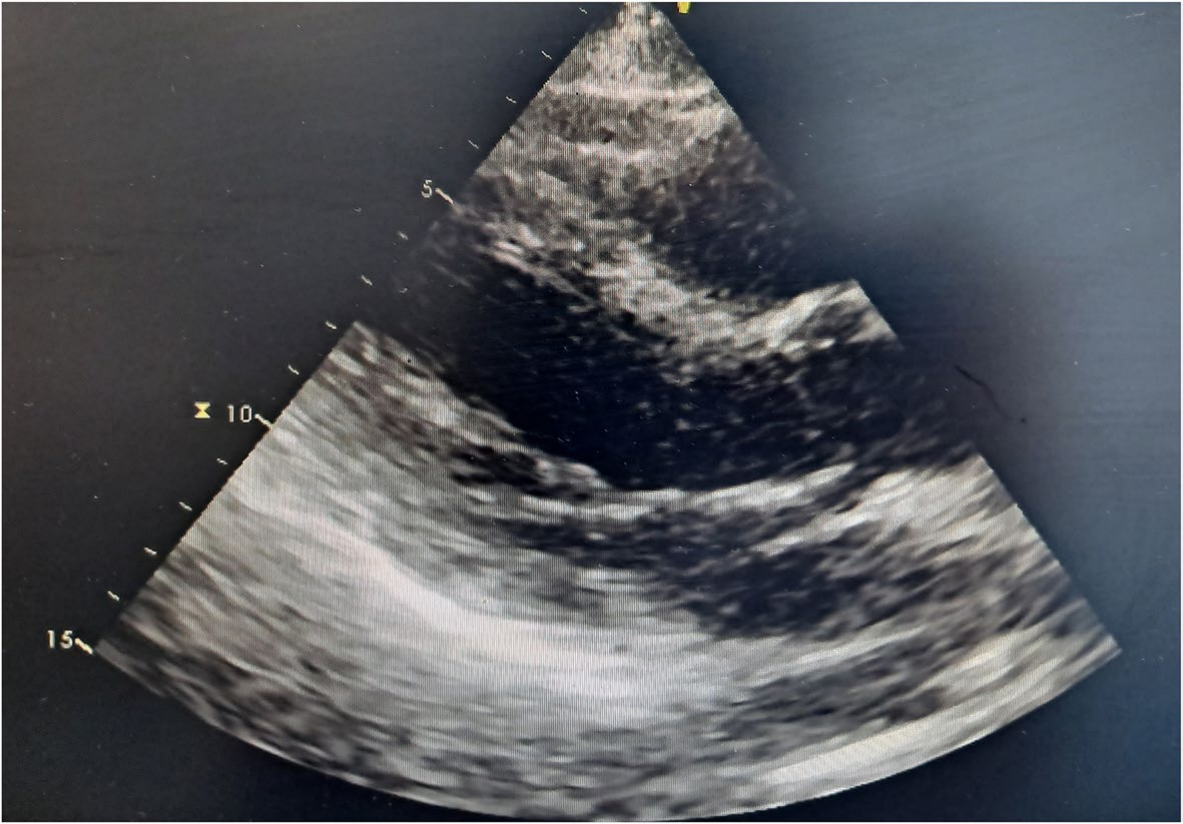
The echocardiography after tislelizumab therapy in August 2021. Compared to that of April 2021, the left atrial mass vanished

At that time, the patients had failed to respond to first-line chemotherapy, according to China’s National Medical Products Administration (NMPA) in 2020, we decided to try the tislelizumab immunotherapy. After obtaining the patient's consent, he received 6 cycles of systematic tislelizumab immunotherapy (200 mg every 3 weeks) between May and September 2021, with minor adverse effects. The re-examination of chest CT in August 2021 showed that the volume of enlarged lymph nodes under the right hilum and tracheal carina was significantly smaller than that in April 2021 (Fig. 5). Brain MRI revealed that, when compared to April 2021, the patient's right temporal lobe lesions were greatly reduced, his bilateral frontal lobe lesions vanished, and his left cerebellar hemisphere lesions were stable (Fig. 6). The resolution of the left atrial mass was confirmed by echocardiography (Fig. 7), and the patient’s dizziness and headache vanished following immunotherapy, with no recurrence of symptoms of loss of consciousness or arrhythmia. From August 2021 to March 2022, the patient received another 6 cycles of tislelizumab immunotherapy (200 mg every 3 weeks). The brain MRI in March 2022 revealed no significant change in head metastasis compared with August 2021. At present, the patient is followed up in the hospital every 3 weeks, and except for an occasional mild headache, his daily life keeps unaffected.

## Discussion

Tislelizumab has been approved by the China NMPA as a treatment for nine indications [16], including relapsed or refractory classical Hodgkin lymphoma (R/R cHL), locally advanced or metastatic urothelial carcinoma (UC) after failure of platinum-based chemotherapy, squamous non-small cell lung cancer (NSCLC), driver gene negative non-squamous NSCLC, hepatocellular carcinoma (HCC), non-small cell lung cancer (NSCLC), previously treated microsatellite instability-high (MSI-H) or mismatch repair gene-deficient (dMMR) advanced stage solid tumors, esophageal squamous cell carcinoma (ESCC), and nasopharyngeal carcinoma (NPC). Although tislelizumab is not approved for use outside of China, it has been submitted for regulatory review as a potential treatment for unresectable recurrent locally advanced or metastatic ESCC after prior systemic therapy in the USA, and in NSCLC and ESCC in Europe.

Bladder cancer is a common tumor of genitourinary system, with a median diagnosis age of slightly more than 70 years [17]. Advanced age and medical comorbidities might pose the question of whether treatment should be recommended or not. In a study of 126 patients with muscle-invasive bladder cancer (64 patients did not receive any definitive local treatment, 62 patients received radical cystectomy or radiation therapy), Martini et.al. revealed that untreated patients had an increased risk of progression to metastatic disease (hazard ratio [HR] 2.40, 95% CI 1.28, 4.51; *P* = 0.006), death from any cause (HR 2.63, 95% CI 1.65, 4.19; *P* < 0.001) and cancer-specific mortality (subdistribution HR 2.02, 95% CI 1.24, 3.30; *P* = 0.004) [18]. Thus, the appropriateness of treatment should always be discussed, given the extremely poor prognosis if the disease is left untreated.

There are two primary routes of bladder cancer metastasis: one is via the lymphatic drainage system to regional lymph nodes, and the other is via the bloodstream to metastasis to distant organs. Tumor-associated lymphangiogenesis, tumor cell epithelial-mesenchymal transition (EMT), and tumor microenvironment are the key processes of lymph node metastasis [19]. The most frequent sites of metastases were regional lymph nodes (90%), liver (47%), lung (45%), bone (32%), peritoneum (19%), pleura (16%), kidney (14%), adrenal gland (14%), and the intestine (13%) [3], whereas brain metastases are uncommon [4], and cardiac metastasis has not been reported.

Due to the capacity of the blood-brain and blood-tumor barriers to protect tumor cells from systemic chemotherapeutic medicines, the brain serves as a sanctuary site for metastatic tumor cells [20]. Sternberg et al. reported in 1989 that MVAC (methotrexate, vinblastine, doxorubicin, cisplatin) treatment resulted in brain metastases in 16% of 121 patients with advanced bladder transitional cell carcinoma (TCC) [21]. Dhote et al. reported in 1998 on 50 patients with advanced TCC treated with the MVAC chemotherapy regimen, 8 (16%) of whom developed brain metastases [22]. They discovered that brain metastases from bladder cancer frequently emerge late in the disease course, possibly due to MVAC medications' inability to cross the blood-brain barrier, increasing the risk of brain metastases. Diamantopoulos et al. also believed that, due to the protective effect of the blood-brain barrier, the central nervous system may favor the development of dormant and particularly drug-resistant malignant cells, resulting in tumor metastasis in the central nervous system [5]. Sarmiento et al. described a patient who developed solitary brain metastases from transitional cell carcinoma 14 years after receiving gemcitabine for bladder cancer, implying that chemotherapeutic agents such as gemcitabine, which can cross the blood-brain barrier, may delay the development of brain metastases [23]. Rosiello et al. investigated 5767 patients with metastatic bladder cancer in the USA and concluded that, unlike lung, bone, and other organ metastases, central nervous system metastases are not associated to race or age [24].

Brain metastases are primarily exhibited clinically as nausea, vomiting, headache, ataxia, disorientation, seizures, and loss of consciousness, as well as focal neurological symptoms (hemiplegia, blindness, cranial nerve palsy) and meninges due to meningeal carcinomatosis [5]. Because brain metastases are uncommon in bladder cancer patients and head CT or MRI are not routinely performed, doctors should be aware of the possibility of brain metastases in patients presenting with neurological symptoms and complete head CT or MRI promptly. The current standard of care for brain metastases consists mostly of surgery, systemic chemotherapy, and whole brain or stereotactic irradiation, which may be administered alone or in combination [5]. In a retrospective study of 16 patients with brain metastases from bladder cancer, surgery was found to be superior to radiotherapy alone [6]. Another study of 62 patients with bladder cancer who developed brain metastases revealed no significant difference in overall survival or intracerebral control between surgical resection with radiation therapy and radiation therapy alone [25]. While Rades et al. found no statistically significant difference between whole-brain irradiation plus a boost to the metastatic site and whole-brain irradiation plus radiosurgery in bladder cancer patients with solitary brain metastases, the former may be a better treatment option due to its less invasiveness [26]. At present, there is still no consensus on the treatment of bladder cancer patients with brain metastases. Patients treated with chemotherapy had a median survival of 2–4 months, and patients with multiple brain metastases had a worse prognosis than patients with single brain metastases [6, 7].

The incidence of cardiac metastases in cancer patients exceeds 10%, which is significantly greater than the incidence of primary heart malignancies [27, 28]. Hematogenous metastases are common in melanoma, lymphoma, and sarcoma, which can lead to myocardial and endocardial involvement. Lymphoid metastases are common in lung cancer and can result in pericardial and epicardial involvement. Metastasis to the right atrium via the inferior vena cava is common in renal cancer, liver cancer, uterine leiomyoma and pheochromocytoma. Direct invasion can be seen in locally invasive tumors such as mediastinal and pleural tumors and breast cancer [29]. The most common tumors that metastasize to the heart are lung cancer (37%), breast cancer (7%), esophageal cancer (6%), and hematological malignancies such as lymphoma (20%) [30]. Because bladder cancer heart metastases have not previously been described, our case is likely to be the first, which may be disseminated through blood.

Over 90% of secondary cardiac tumors are clinically asymptomatic and are typically discovered until after death [31]. Patients may present with nonspecific symptoms, distant embolism symptoms, or direct tumor invasion symptoms, such as blockage, cardiac tamponade, and arrhythmias [32]. Clinicians should be aware of the risk of cardiac metastases in cancer patients who have new-onset cardiac symptoms and should choose further examinations, such as echocardiography, CT, or MRI, based on individual circumstances. Cardiovascular metastases have a 5-year survival rate of 26% in patients with advanced malignancies, and while the goal of treatment is to alleviate symptoms and prevent tumor recurrence, outcomes are usually unsatisfactory [33]. For cardiac tamponade, immediate pericardiocentesis is required, followed by topical chemotherapy drugs or radioisotopes to prevent recurrence [34]. For life-threatening arrhythmias, antiarrhythmic drugs should be used first, and radiofrequency ablation should be performed for uncontrolled arrhythmias [35]. Due to the generally poor prognosis associated with cardiac metastases, radical surgery is rarely considered. Miralles et al. performed complete and incomplete resection for 2 patients with metastatic cardiac cancer, but died 1 month and 5 days after surgery, respectively [36]. However, for patients with intracardiac obstruction, a positive prognosis, a resectable tumor, and no metastases to other sites, surgery is the preferred therapeutic option [33]. Furthermore, in some cases, chemoembolization of a single heart mass supplied by coronary arteries may be advantageous [37].

Previous studies have demonstrated that second-line ICI treatment can significantly improve patient survival and may even be more effective in patients with high PD-L1 expression [9–13]. A recent meta-analysis revealed that PD-1 or PD-L1 inhibitors can reduce risk of both disease progression and death of patients with brain metastases of NSCLC [38]. The clinical trial CTR20170071 enrolled 104 Asian patients with locally advanced or metastatic urothelial cancer who had previously received platinum-containing chemotherapy. The results indicated that tislelizumab treatment had a 24.8% objective response rate (ORR) and a 38.6% disease control rate (DCR) [15]. However, despite the fact that the majority of patients (76%) in this trial had visceral metastases, including 24% with liver and 23% with bone metastases, no patients with brain or heart metastases were included. In this study, we report the first case of tislelizumab being beneficial for urothelial carcinoma with brain and heart metastases. Despite receiving first-line chemotherapy, we think that surgically treated patients with cerebral and intracardiac lesions have a poor prognosis. Given Tislelizumab’s remarkable efficacy in the treatment of advanced bladder cancer, it was decided to begin systemic immunotherapy after consulting with the doctor and obtaining patient consent. After 6 cycles of systematic tislelizumab immunotherapy, re-examination revealed that the patient’s right temporal lobe lesions significantly decreased, bilateral frontal lobe lesions vanished, left cerebellar hemisphere lesions stabilized, the left atrial mass resolved, and the dizziness and headache resolved, with no recurrence of symptoms of loss of consciousness or arrhythmia. It has been 16 months since the patient was diagnosed with brain metastasis and heart metastasis of bladder cancer by the time this article was written, and the general condition of the patient is still good. His survival time has far exceeded the documented general survival time of brain metastatic cancer patients undergoing surgery or chemotherapy, and he also survived far longer than heart metastatic cancer patients undergoing surgery. We believe that the benefit of tislelizumab immunotherapy may outweigh the benefit of surgical excision of metastases, especially head and heart metastases, for which the effect of chemotherapy, radiotherapy and surgery is poor, but more study is needed to validate this.

Additionally, unlike other anti-PD-1 antibodies, tislelizumab was specifically engineered to minimize Fcγ receptor binding to limit antibody-dependent phagocytosis, a mechanism of potential resistance to anti–PD-1 therapy [39, 40]. We speculate that this property contributes to its inhibitory effect on distant metastasis. However, we cannot make sure whether earlier tislelizumab treatment would have been more effective at preventing tumor metastasis in this patient. To maximize the benefits for bladder cancer patients, we anticipate more clinical trials evaluating early-stage and neoadjuvant/adjuvant immunotherapy will be conducted in the future.

## Conclusion

Although brain and cardiac metastases from bladder cancer are uncommon, clinicians should be cautious and do further examinations in individuals who present central nervous system or cardiac symptoms. These individuals have a poor prognosis, and the primary purpose of treatment is to alleviate symptoms. To maximize the benefits for bladder cancer patients, we anticipate more clinical trials evaluating early-stage and neoadjuvant/adjuvant immunotherapy will be conducted in the future.

## Data Availability

The datasets generated and analyzed during the present study are available from the corresponding author on reasonable request.
